# Pancreatic Enzyme Supplements Are Not Effective for Relieving Abdominal Pain in Patients with Chronic Pancreatitis: Meta-Analysis and Systematic Review of Randomized Controlled Trials

**DOI:** 10.1155/2016/8541839

**Published:** 2016-04-24

**Authors:** Mohammad Yaghoobi, Julia McNabb-Baltar, Raheleh Bijarchi, Peter B. Cotton

**Affiliations:** ^1^Division of Gastroenterology, McMaster University, Hamilton, ON, Canada; ^2^Division of Gastroenterology, Hepatology and Endoscopy, Brigham and Women's Hospital, Harvard Medical School, Boston, MA, USA; ^3^Division of Respirology, St Michael's Hospital, Toronto, ON, Canada; ^4^Division of Gastroenterology and Hepatology, Medical University of South Carolina, Charleston, SC, USA

## Abstract

*Background*. Pancreatic enzyme supplementation is widely used to treat pain in patients with chronic pancreatitis, despite little evidence for efficacy. We performed a systematic review of the literature and a meta-analysis to investigate its effectiveness.* Methods*. All randomized controlled parallel or crossover trials in patients with chronic pancreatitis comparing pancreatic enzyme supplementation to placebo were included. The main outcome was improvement in pain score or reduced analgesic consumption. Two independent reviewers extracted data. Mantel-Haenszel random effect model meta-analysis was used whenever methodologically appropriate.* Results*. Five out of 434 retrieved studies were included in the systematic review. All studies used relatively similar methodology. Four studies using enteric-coated pancreatic enzyme supplementation failed to show any improvement in pain as compared to placebo. The only study using non-enteric-coated enzymes did show reduction in the pain score. There was significant heterogeneity among studies in both analyses. Random model meta-analysis of three studies showed no significant difference in the mean of daily pain score (mean difference: 0.09 (1.57–1.39), *p* = 0.91) or average weekly analgesic consumption (mean difference: −0.30 (−2.37–1.77), *p* = 0.77) between the periods of administering pancreatic enzyme supplementation versus placebo.* Conclusion*. Pancreatic enzyme supplements do not seem to relieve abdominal pain in patients with chronic pancreatitis and should not be prescribed solely for this purpose, given their significant cost and potential side effects.

## 1. Introduction

Abdominal pain is a major complaint and difficult to manage, in patients with chronic pancreatitis [1]. Pancreatic enzyme supplementation (PES) has been used extensively despite lack of strong evidence for its efficacy, and the medications are expensive.

The rationale for using PES is simple and logical. It should inhibit the release of cholecystokinin and secretin and thereby decrease the secretion and potential premature activation of pancreatic enzymes in the pancreatic ducts, which is believed to contribute to pancreatic pain [2].

However, this theory has not been proven. There have been a few clinical trials (with significant heterogeneity in their results), but, to our knowledge, there have been no large randomized clinical trials or meta-analyses. Therefore, we performed a systematic review of the literature and a meta-analysis of relevant published randomized clinical trials comparing PES with placebo in treating abdominal pain in patients with chronic pancreatitis.

## 2. Material and Methods

### 2.1. Sources

Two independent reviewers performed the search, risk of bias assessment, and data extraction (Mohammad Yaghoobi and Julia McNabb-Baltar). A third reviewer (RB or Peter B. Cotton) was involved when a consensus could not be achieved. Electronic searches were conducted using OVID MEDLINE (1946 to January 2015), EMBASE (1980 to January 2015), Cochrane library, and ISI Web of knowledge from 1980 to January 2015. Articles were selected using a highly sensitive search strategy, with a combination of MeSH headings and text words that included (i) chronic pancreatitis, (ii) enzymes, and (iii) pain. Recursive searches and cross-referencing were carried out using “similar articles” function. Bibliography of the articles identified after an initial search was also manually reviewed. The search was not restricted to any specific language (Figures 2 and 3).

### 2.2. Study Selection

Randomized controlled parallel or crossover trials were included. Included patients were those with chronic pancreatitis. The intervention was pancreatic enzyme supplementation and the control was identical placebo. The outcome of interest was improvement in pain score or reduction in analgesic consumption.

Studies with missed or nonextractable data, studies in children, abstracts, and duplicate publications were excluded. No restrictions were applied in terms of language, geographical location, or quality of studies.

### 2.3. Heterogeneity

Variation in the patient populations and the quality of studies was considered as* a priori* source of heterogeneity. Subgroup analyses were predicted* a priori* to investigate each source; however, no subgroup analysis could be performed due to insufficient data.

### 2.4. Quality Assessment

Two reviewers retrieved the data. The methodological quality of studies was assessed by using Cochrane Collaboration tool for assessing the risk of bias [3]. No studies were excluded based on the quality score.

### 2.5. Statistical Analysis

Whenever at least two studies used similar method in reporting the outcome, we planned to perform meta-analysis of using the Mantel-Haenszel method and Review Manager 5.0.25. The random effects model was applied since significant heterogeneity was predicted. A *p* value of less than 0.05 was used as criterion for statistical significance. *I* ^2^ was generated to assess heterogeneity and was interpreted as previously described [4]. Test of heterogeneity was considered significant if the *p* value was less than 0.10. All results are reported with 95% confidence intervals (CI) when applicable. Sensitivity analyses were planned based on the weight of the trials and by excluding each individual trial in turn as recommended by Cochrane Collaboration open learning material for reviewers [5].

## 3. Results

**Figure 1:**
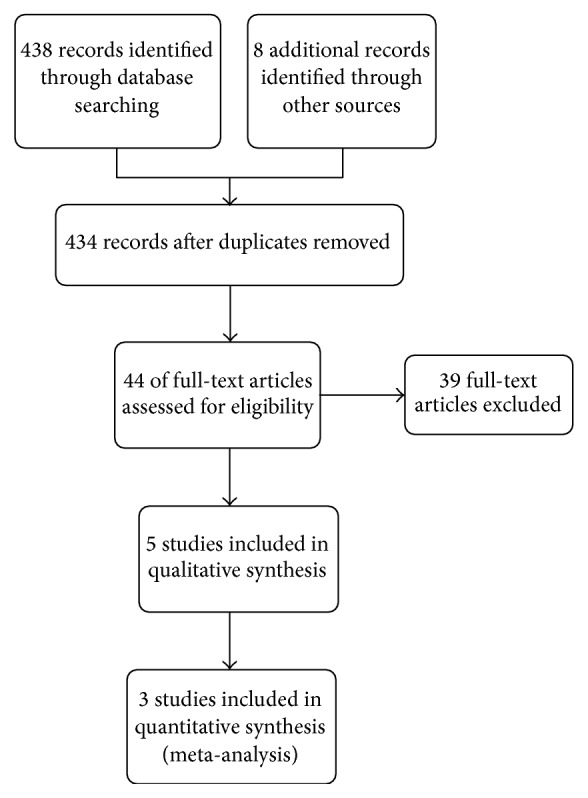
The PRISMA flow diagram of study selection process.

**Figure 2:**
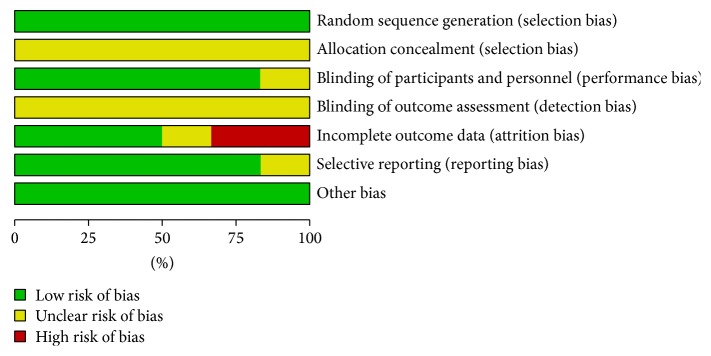
Risk of bias graph.

**Figure 3:**
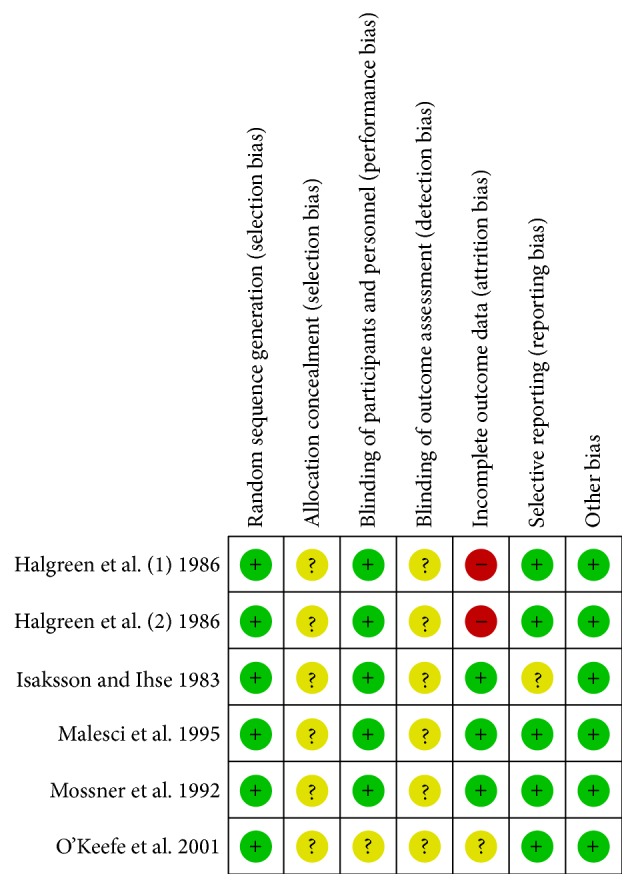
Consensus risk of bias assessment of the included studies. Green: low risk, yellow: unclear, and red: high risk.

Five out of 434 retrieved studies were eligible and were included in the systematic review.  Figure 1 depicts the PRISMA flow diagram.  Table 1 depicts the characteristics of included studies. Four studies were designed as crossover double-blind randomized controlled trials and one as a parallel double-blind randomized controlled trial. Four studies used enteric-coated enzymes and one non-enteric-coated ones [7]. One study included eight postsurgical patients [7]. All studies used relatively similar methodology but reported different outcomes. Only two studies used a similar scale to report mean pain scores and therefore a meta-analysis was done on these two studies [6, 8]. Moreover, two studies reported average analgesic consumption similarly, thus being appropriate to be included in a meta-analysis [7, 8].

### 3.1. Pain Alleviation

Four studies using enteric-coated pancreatic enzyme supplementation failed to show any improvement in pain as compared to placebo.

The only study which showed improvement in pain score was the oldest study and the only one using non-enteric-coated enzymes [7]. This randomized crossover Swedish study involved 19 patients with chronic pancreatitis (based on the low pancreatic isoamylase in serum, abnormal Lundh test, calcifications on plain X-ray, endoscopic retrograde cholangiopancreatography (ERCP) findings, or operative and histological findings), including eight who previously underwent Puestow or duVal pancreatojejunostomy or subtotal pancreatectomy. They randomly received one-week enzyme supplementation or placebo preceded by a wash-out period of one week. At the end of the study, 10 had responded significantly to enzyme supplementation as compared with 9 who did not respond. 15 patients reported subjective pain relief during the week of active treatment as compared to the week of placebo (*p* < 0.05). Each patient reported an average of 30% pain reduction (*p* < 0.01). The median analgesic consumption was not significantly different between two groups (7.8 versus 8.9 tablets per week with enzymes versus placebo, resp.).

In another 4-week double-blind randomized crossover study, 20 Danish patients [11 with (Halgreen et al., (1)) and 9 without steatorrhea (Halgreen et al., (2))] with chronic pancreatitis were included, based on a reduced exocrine pancreatic function using Lundh test and at least one of the pancreatic calcifications, previous acute attacks of pancreatitis, and/or typical abnormalities by ERCP [6]. Each patient randomly received either enzymes or placebo for 2 weeks followed by the other preparation for another 2 weeks. Pain was measured based on a 10 cm linear analogue scale (0–10). The results showed that none of the postprandial pain scores, pain scores between meals, number of pain attacks, analgesic consumption, subjective pain scores, or general well-being were significantly different in two groups.

In another prospective crossover placebo-controlled double-blind multicenter study from Germany, 47 patients with chronic pancreatitis (based on abdominal ultrasound, ERCP, and abdominal CT) were included to receive porcine pancreatic extracts or placebo. After two weeks there was no significant difference between the two groups with regard to pain score or analgesic consumption.

Malesci et al. reported a double-blind randomized crossover study in 26 Italian patients with chronic pancreatitis based on clinical presentation and the presence of ductal changes at ERCP, pancreatic calcifications, abnormalities at ultrasonography, and pancreatic insufficiency at the secretin-cerulein test [8]. They were randomly assigned to 4-week therapy with enzymes or placebo followed by 4 weeks of alternate therapy. The pain was measured using a 10 cm linear visual analogue scale (0–10). Four patients left or were withdrawn from the study. In the 22 patients who ended the trial, the 4-month cumulative score, number of days and hours with pain, and the number of long-lasting (>12 h) pain attacks were not statistically different in two groups. The median analgesic consumption, adjusted for drug potency, was also not statistically different.

A newer randomized parallel-group study from South Africa included patients with chronic pancreatitis based on suppressed cholecystokinin-stimulated enzyme secretion or steatorrhea and evidence of chronic pancreatitis in CT or ultrasound scanning, ERCP, or the presence of pancreatic calcification on abdominal X-ray. They were included in a one-week placebo run-in period [10]. Among those, 29 patients who malabsorbed more than 10 g of fat per day were randomized to receive 14 days of either enzymes or placebo. Overall, supplementation had no significant effect on the severity of abdominal pains or distension.

### 3.2. Meta-Analyses

#### 3.2.1. Mean Daily Pain Score

**Figure 4:**
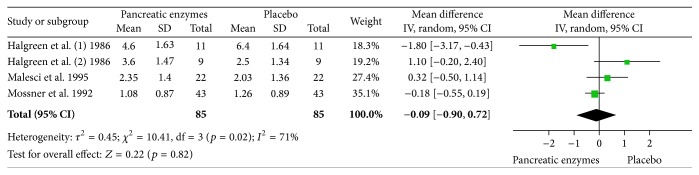
Random model meta-analysis of the mean of daily pain score in two crossover studies (*n* = 42).

Two studies reported mean daily pain scores based on a 10 cm linear analogue scale [6, 8]. Halgreen et al. reported the results separately for those with or without steatorrhea. Malesci et al. reported cumulative pain score over the course of the study and we had to calculate daily mean pain scores from the data in order to have consistent information from these two studies to perform the meta-analysis. Random model meta-analysis of these two crossover studies including 42 patients showed no significant difference in the mean of daily pain score between the periods of administering pancreatic enzyme supplementation versus placebo (mean difference: 0.09 (1.57–1.39), *p* = 0.91). There was significant heterogeneity among studies as expected from the sample size and methodology (*p* = 0.007, *I* ^2^: 80%).  Figure 4 depicts the forest plot of this analysis. The results remained unchanged in sensitivity analysis after exclusion of each trial in turn.

#### 3.2.2. Analgesic Consumption

**Figure 5:**
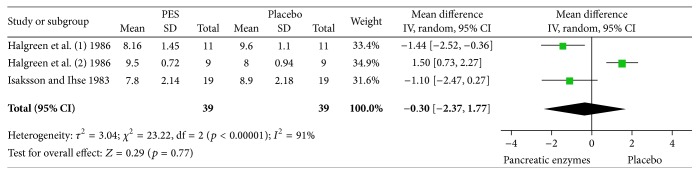
Random model meta-analysis of the average analgesic consumption per week in two crossover studies (*n* = 39).

Two studies reported average weekly analgesic consumption [6, 7]. Halgreen et al. reported the results separately for those with or without steatorrhea. Random model meta-analysis of these two crossover studies including 39 patients showed no significant difference in the average weekly analgesic consumption between the periods of administering enzymes versus placebo (mean difference: −0.30 (−2.37–1.77), *p* = 0.77). There was significant heterogeneity among studies as expected from the sample size and methodology (*p* < 0.00001, *I* ^2^: 91%).  Figure 5 depicts the forest plot of this analysis. The results remained unchanged in sensitivity analysis after exclusion of each trial in turn.

## 4. Discussion

To our knowledge, this is the first comprehensive systematic review and meta-analysis specifically done to address the value of enzyme supplementation in relieving pain in patients with chronic pancreatitis and reports a meta-analysis of subjective pain scores. This systematic review and meta-analysis provided further evidence for the lack of effectiveness in using enzyme supplementation in the management of pain in patients with chronic pancreatitis.

The only previous meta-analysis done on this topic was done by Brown et al. in 1997 [11]. They looked at patient's preference in choosing enzyme supplements versus placebo. The pooled percentage of patients per study who preferred pancreatic enzyme to placebo was 52% ((45%–60%), *p* = 0.52). They did not report any data on pain scores among studies. However, their conclusion is consistent with what we found in our study.

Only one small old study using non-enteric-coated pancreatic enzyme supplements showed improvement in pain as compared to placebo [7]. However, it has one major methodological concern of possible selection bias, since 40% of the included patients had previously undergone surgery and the authors did not report subgroup data for those who had not have surgery. Therefore, it would be cautious not to generalize the results to patient with surgically intact pancreas. One can also argue that the different results of this study might be explained by the use of non-enteric-coated as compared to enteric-coated form of enzyme supplementation. Enteric-coated enzyme supplements are released in the mid-small bowel and therefore may not effectively suppress the feedback loops regulating release of cholecystokinin that occurs in duodenum [2]. This theoretical advantage of non-enteric-coated over enteric-coated forms has not been proved in any controlled clinical trials. Even if this was true, the result of this small study could not be generalized to daily practice, since pancreatic enzyme supplements prescribed in the US are almost exclusively enteric-coated.

A Cochrane review on the role of pancreatic enzyme supplements in chronic pancreatitis used several outcomes, including pain and analgesic consumption [12]. They concluded that enzyme supplements were not beneficial in reducing pain or steatorrhea in patients with chronic pancreatitis. However, the study failed to perform any meta-analysis since the reported outcomes in their included studies were not similar.

One drawback of our study relates to the nature of systematic review and meta-analysis. The possibility of missed trials cannot be completely ruled out. We minimized this possibility by including several types of publications and search methods. Our study did not include any patient-level data analysis due to insufficient reported data and therefore is unable to characterize potential individual predictors of response to enzyme supplementation. If such data were available, it would help in predicting patients who may see pain relief from the therapy. The other main limitation of our study is the heterogeneity among the included studies. All studies included in the meta-analysis were published more than two decades ago and the authors did not necessarily follow the current standards in reporting methodology and results. This significantly contributed to the observed heterogeneity. Despite this, the main results of each meta-analysis confirmed the conclusion from each individual included study. Moreover, to minimize the effect of heterogeneity we used random effects model rather than a fixed effects model meta-analysis [5].

The result of this study should be interpreted with caution given the poor quality of included studies. A large randomized controlled trial comparing PES and identical placebo in patients with chronic pancreatitis will be optimal to address this question. Despite lack of evidence on effectiveness of enzyme supplements and their significant cost, many gastroenterologists still use them. Our findings indicate that this practice should be discouraged.

## Supplementary Material

Characteristics of included studies.

## Figures and Tables

**Table 1:** Characteristics of five included studies.

Study	Year	Country	Method	Pancreatic enzyme	Duration
Halgreen et al. [6]	1986	Denmark	Double-blind randomized crossover	Enteric-coated Pancrease (lipase 20,000, amylase 20,000, and protease 25,000), two capsules at meals and one capsule with snacks	2 weeks

Isaksson and Ihse [7]	1983	Sweden	Double-blind randomized crossover	Pankreon (granulated pancreatic enzymes)	One week

Malesci et al. [8]	1995	Italy	Double-blind randomized crossover	Enteric-coated Pancrex-Duo (lipase 13,000 UI, amylase 43,570 UI, and protease 34,375 IU)	4 months

Mossner et al. [9]	1992	Germany	Double-blind randomized crossover	Enteric-coated Panzytrat (20,000 IU; 5 × 2 capsules/day; proteases/capsule 1,000 IU)	2 weeks

O'Keefe et al. [10]	2001	South Africa	Randomized, parallel group	Enteric-coated capsules (four capsules with meals, two with snacks; content/capsule: lipase 10,000 USP units, protease 37,500 units, and amylase 33,200 units)	2 weeks
